# Macroscopic Internal Variables and Mesoscopic Theory: A Comparison Considering Liquid Crystals †

**DOI:** 10.3390/e20010081

**Published:** 2018-01-22

**Authors:** Christina Papenfuss, Wolfgang Muschik

**Affiliations:** 1 Department of Engineering 2, Hochschule für Technik und Wirtschaft Berlin, 12459 Berlin, Germany; 2 Institut für Theoretische Physik, Technische Universität Berlin, 10623 Berlin, Germany

**Keywords:** mesoscopic theory, internal variables, liquid crystals, damage parameter, dipolar media, flexible fibers

## Abstract

Internal and mesoscopic variables differ fundamentally from each other: both are state space variables, but mesoscopic variables are additionally equipped with a distribution function introducing a statistical item into consideration which is missing in connection with internal variables. Thus, the alignment tensor of the liquid crystal theory can be introduced as an internal variable or as one generated by a mesoscopic background using the microscopic director as a mesoscopic variable. Because the mesoscopic variable is part of the state space, the corresponding balance equations change into mesoscopic balances, and additionally an evolution equation of the mesoscopic distribution function appears. The flexibility of the mesoscopic concept is not only demonstrated for liquid crystals, but is also discussed for dipolar media and flexible fibers.

## 1. Introduction

There are two different possibilities to deal with complex materials within continuum thermodynamics: The first way is to introduce additional state space variables which depend on position and time and extend the state space accounting for the internal structure of the complex material. These additional fields can be internal variables [1,2], order or damage parameters [3], Cosserat triads [4,5,6], directors [7,8], alignment and conformation tensors [9,10]. It is also possible to introduce internal variables, without specifying their physical meaning in the beginning (but obviously, the physical meaning of the considered internal variable has to be made clear finally). This has been successfully applied for instance in rheology [11,12,13,14].

The other way is the so called mesoscopic theory whose idea is to enlarge the *domain* of the field quantities beyond position and time by mesoscopic variables. Consequently, the fields—now called mesoscopic fields—are defined on the mesoscopic space $R_{x}^{3}\times R_{t}\times M$. The manifold M is given by the set of mesoscopic variables which represent internal degrees of freedom depending on the internal structure of the complex material under consideration.

Beyond the additional mesoscopic variables m∈M which belong to each particle in a volume element around x at time *t*, the mesoscopic concept introduces a statistical element, the mesoscopic distribution function f(x,t,m) which describes the distribution of m contained in the considered volume element. This distribution function generates the term “mesoscopic” because this concept includes more information than a “macroscopic” theory on $R_{x}^{3}$ $\times \;R_{t}$, but the microscopic level is not considered like in a kinetic theory, molecular dynamics, quantum-theoretical or other “microscopic” approaches. Thus, the mesoscopic level of information is between the microscopic and the macroscopic ones.

The aim of the present paper is to discuss the connection between the macroscopic theory of internal variables on space–time and the mesoscopic theory on the mesoscopic state space. An equation of motion of the internal variables can be derived from macroscopic thermodynamics. However, by starting with the mesoscopic theory, the mesoscopic origin of the internal variable and its equation of motion becomes visible. Obviously, the mesoscopic distribution function cannot be determined by only one macroscopic internal variable: it is determined by all its (infinity of) moments [15]. Because only a finite set of macroscopic variables is available, the reconstruction of the mesoscopic distribution function is only possible within a certain restricted class of functions, namely the distribution functions maximizing the entropy under the constraint of a prescribed value of certain moments. In the following, we will investigate the relation between an internal variable theory and a mesoscopic one by considering the example of liquid crystals and some other mesoscopic items.

## 2. Fundamental Balances and Basic Fields

We consider here a special part of the realm of non-linear field theories of classical physics, especially Continuum Thermodynamics [16] whose aim is the determination of the wanted (or basic) fields which obey balance equations. In continuum mechanics, these seven basic fields are the mass density ϱ, the velocity v of the material and its spin density s

(1) $$B_{mech}\left(x,t\right)\;=\;\left(\varrho ,v,s\right)\left(x,t\right).$$

The domain of these fields is the non-relativistic space–time. Seven balance equations belong to these seven basic fields: the mass balance, the momentum and the spin balance. Constitutive fields appear in them: the stress tensor T and the couple stress W. Momentum supply ϱk and spin supply ϱg are externally given quantities.

Two basic fields are added to Continuum Mechanics to obtain Continuum Thermodynamics: the densities of internal energy *e* and entropy η

(2) B(x,t)=(ϱ,v,s,e,η)(x,t).

The heat flux q and the entropy flux Φ are the additional constitutive fields. The corresponding external supplies are the internal energy supply ϱr and the entropy supply ϱγ. If constitutive equations are not presupposed, a balance equation of the temperature *T* does not exist: temperature can be defined by T:=r/γ.

The constitutive fields of simple Continuum Thermodynamics

(3) R(x,t)=(T,W,q,Φ,s,η)(x,t)

do not only depend on basic fields (2), but also on their derivatives, as the ‘Fourier law of heat conduction’ q(ϱ,T,∇T)=−κ(ϱ,T,∇T)∇T shows (Here, the internal energy is replaced by the temperature.). Fourier’s law demands, that we have to introduce a domain of the constitutive fields Z(x,t) which also contains derivatives of the basic fields. We call this domain *state space* or *constitutive space*. The most simple state space is that of a fluid without internal friction and missing heat conduction which contains the mass density and the internal energy

(4) Z(x,t)=(ϱ,e)(x,t).

The velocity v does not occur in state spaces because the relative velocity between material and observer does not influence constitutive properties (Especially, we consider acceleration-insensitive materials which do not need a so-called “second entry” [17].) in contrast to ∇v on which the stress tensor may depend.

Additional internal friction and heat conduction makes a state space necessary which contains the spatial derivatives of mass density, internal energy and velocity

(5) Z(x,t)=(ϱ,e,∇ϱ,∇e,∇v)(x,t).

Aging processes need additionally time derivatives

(6) $$Z\left(x,t\right)\;=\;\left(\varrho ,e,\nabla \varrho ,\nabla e,\nabla v,\overset{{}_{{}^{•}}}{\varrho },\overset{{}_{{}^{•}}}{e},\overset{{}_{{}^{•}}}{v}\right)\left(x,t\right).$$

According to (3), we obtain the following scheme for the representation of constitutive properties

(7) $$R\left(Z(x,t)\right)\;=\;\left(T,W,q,\Phi ,s,\eta \right)\left(Z(x,t)\right).$$

This means, constitutive properties depend on the space–time via the space–time dependence of the state space variables, and the derivatives ∇ and $\partial_{t}$ need a state space and have to be performed by use of the chain rule.

Considering the examples (4)–(6), the state space (4) is extended by derivatives of basic fields. Obviously, other extensions of a state space taking other than the basic fields into account are possible resulting in state spaces which belong to so-called complex materials.

## 3. Complex Materials

Complex materials are characterized by a state space which contains variables beyond the basic fields and their derivatives. A famous example for such a state space is that of the Extended Thermodynamics. Other examples of extended state spaces are those belonging to thermoviscoelastic and thermoviscoplastic materials and materials showing thermal after-effects.

### 3.1. Extended Thermodynamics

The extended state space of Extended Thermodynamics is [18,19]

(8) Z(x,t)=(ϱ,e,T+p1,q)(x,t).

The original state space is extended by the constitutive quantities, here the viscous part of the stress tensor and the heat flux density, which are now on equal foot in the state space with mass density and internal energy. In Extended Thermodynamics, the state space (4) is extended by well defined fields. Another possibility of extension is the introduction of presently undefined variables as place-holders defining them later. Such variables are specified as *internal*.

### 3.2. Internal Variables

Historically, the concept of internal variables can be traced back to Bridgman [20], Meixner [21], and many others. The introduction of internal variables makes possible the use of large state spaces, that means, material properties can be described by mappings defined on the state space variables (including the internal ones), thus avoiding the use of their histories which appear in small state spaces [1]. Those are generated, if the internal variables are eliminated. Consequently, internal variables allow to use the methods of Irreversible and/or Extended Thermodynamics [22].

Internal variables cannot be chosen arbitrarily: there are concepts which restrict their introduction [1]. The most essential ones are:

1. For the present, internal variables can be introduced as place-holders for elucidating the considered constitutive structure, but finally, they need a model or an interpretation.
2. Beyond the constitutive and the balance equations, internal variables require rate equations which can be adapted to different situations, making their use flexible and versatile.
3. The internal variables and their time rates do not occur in the balance equation of the internal energy.
4. A local isolation does not influence the internal variables locally.
5. In equilibrium, the internal variables become dependent on the variables of the equilibrium sub-space.

Satisfying these concepts, the internal variables entertain an ambiguous relation with constitutive microstructure [2]. A state space extended by internal variables is e.g.,

(9) Z(x,t)=(ϱ,e,∇ϱ,∇e,∇v,ξ)(x,t),

and the evolution equations may have the shape

(10) $$\overset{{}_{{}^{•}}}{\xi }\;=\;f\left(⊗\right)+g\left(⊗\right)\overset{{}_{{}^{•}}}{e}+\;h\left(⊗\right)\cdot \nabla e+k\left(⊗\right)\cdot \nabla v,\;⊗\;=\;\left(\varrho ,e,\nabla \varrho ,\nabla e,\nabla v,\xi \right).$$

Special one-dimensional cases are

(11) $$\begin{array}{cc} \mathrm{relaxation}\;\mathrm{type}: & \;\overset{{}_{{}^{•}}}{\xi }\left(t\right)\;=\;-\frac{1}{\tau (⊗)}\left(\xi \left(t\right)-\xi^{eq}\right), \end{array}$$

(12) $$\begin{array}{cc} \mathrm{reaction}\;\mathrm{type}\left[1\right]: & \;\overset{{}_{{}^{•}}}{\xi }\left(t\right)\;=\;\gamma \left(⊗\right)\left[1-\exp\left(-\mu (t)\beta (⊗)\right)\right]. \end{array}$$

If Condition 3 is not satisfied, that means, if internal variables occur in the balance eequation of the internal energy, these variables of an extended state space are called *internal degrees of freedom*.

### 3.3. The Mesoscopic Theory

As already mentioned in the Section 1, there is another possibility for describing complex materials: Instead of using extended state spaces which modify the constitutive Equation (7), the domain of the basic fields (2) is extended by so-called mesoscopic variables m [16]

(13) $$B_{meso}\left(m,x,t\right)\;=\;\left(\varrho ,v,s,e,\eta \right)\left(m,x,t\right).$$

These mesoscopic variables are on equal footing with the space–time variables resulting in the fact, that the mesoscopic balance equation of the density X defined on

(14) $$\left(\cdot \right)\equiv \left(m,x,t\right)\in M\times R^{3}\times R^{1}$$

is well known

(15) $$\frac{\partial }{\partial t}\;X\left(\cdot \right)+\nabla_{x}\cdot \left[v(\cdot )\;X(\cdot )-S(\cdot )\right]+\nabla_{m}\cdot \left[u(\cdot )\;X(\cdot )-R(\cdot )\right]=\Sigma \left(\cdot \right).$$

Here, the independent field u(·), defined on the mesoscopic space, describes the change in time of the set of mesoscopic variables: With respect to m the *mesoscopic change velocity* u(·) is the analogue to the mesoscopic material velocity v(·) referring to x: If a particle is characterized by (m,x,t), then for Δt→+0 it is characterized by (m+u(·)Δt,x+v(·)Δt,t+Δt). Besides the usual gradient $\nabla_{x}$ the gradient $\nabla_{m}$ with respect to the set of mesoscopic variables also appears. The non-convective fluxes are S(·) and R(·), supply and production are collected in Σ(·).

Beyond the use of additional mesoscopic variables m, the mesoscopic concept introduces a statistical element, the so-called *mesoscopic distribution function* (MDF) f(m,x,t) generated by the different values of the mesoscopic variable in a volume element

(16) f(m,x,t)≡f(·).

The MDF describes the distribution of m in a volume element around x at time *t*, and therefore it is normalized

(17) ∫f(m,x,t)dM=1.

Now, the fields such as mass density, momentum density, etc. are defined on the mesoscopic space. For distinguishing these fields from the macroscopic ones, we add the word “mesoscopic”. Consequently, the *mesoscopic mass density* is defined by

(18) ϱ(·):=ϱ(x,t)f(·).

Here, ϱ(x,t) is the macroscopic mass density. By use of (17) we obtain

(19) ϱ(x,t)=∫ϱ(m,x,t)dM.

This equation shows, that the system can be formally treated as a mixture of components having the partial density ϱ(·) [23]. Here, the “component index” m is a continuous variable. Because mixture theory is well developed [24,25] *mesoscopic balance equations* can be written down very easily [26]. The special case of liquid crystals is considered in [27].

Other mesoscopic fields defined on the mesoscopic space are the *mesoscopic material velocity* v(·) of the particles belonging to the mesoscopic variable m at time *t* in a volume element around x, the *external mesoscopic acceleration* k(·), the *mesoscopic stress tensor* T(·), the *mesoscopic heat flux density* q(·), etc. Macroscopic quantities are obtained from mesoscopic ones as averages with the MDF as probability density

(20) $$A\left(x,t\right)=\int_{M}A\left(\cdot \right)f\left(\cdot \right)dM.$$

This again shows that the complex material can be seen as a mixture of components with different values of the mesoscopic variable.

## 4. Liquid Crystals

### 4.1. The Macroscopic Theory

#### 4.1.1. General remarks

The molecules of nematic liquid crystals are orientable, which means each molecule has a preferred direction n—the *microscopic director*—that indicates the orientation of the needle-shaped molecule. A particle of the liquid crystal continuum theory contains a lot of molecules of different orientations, resulting in a mean orientation belonging to the considered particle described by a unit vector d. This unit vector—called the *macroscopic director*—is a basic field d(x,t) of the macroscopic director theory of nematic liquid crystals [28,29] (the Ericksen–Leslie theory [27]) whose microscopic background is out of scope (If the microscopic background is taken into account, the Ericksen–Leslie one-director theory allows only parallel or planar orientation of the microscopic directors [30].). As an internal variable, the macroscopic director needs an evolution equation (see Section 4.2.4).The macroscopic director as a basic field does not contain any information about the degree of orientation of the microscopic directors. The same holds true for a macroscopic alignment tensor, which is introduced using the macroscopic director, or as a basic field by its own [9,31] (see Section 4.1.2).

#### 4.1.2. Alignment Tensor as an Internal Variable

In the liquid crystalline state, material properties are anisotropic, in contrast to the isotropic liquid state. On the other hand, liquid crystalline phases behave like fluids, as they do not have a well defined shape but flow like highly viscous fluids. The anisotropic properties of liquid crystals can be described in terms of a second order tensor, the alignment tensor.

A purely macroscopic definition of the alignment tensor in terms of the dielectric tensor reads

(21) $$a:=\frac{\epsilon^{e}-\frac{1}{3}\;\mathrm{trace}\left(\epsilon^{e}\right)\delta }{\frac{1}{3}\;\mathrm{trace}\left(\epsilon^{e}\right)}$$

with the dielectric tensor $\epsilon^{e}$ (D= $\epsilon^{e}\cdot E$).

The second order tensor—defined in Equation (21)—has the following properties:

1. It vanishes in the high temperature phase (the isotropic, ordinary liquid phase), because, in the ordinary liquid phase the dielectric tensor is proportional to the unit tensor δ, and the traceless part vanishes.
2. It is non-zero in the low temperature phase (the nematic liquid crystal phase), because, in this phase, the dielectric tensor has a non-zero traceless part.
3. It is a dimensionless quantity due to the normalization with the trace in the denominator.

With these properties, the second order alignment tensor can be considered as an order paramete in the sense of the Landau-theory of phase transitions. The Landau-theory was developed to deal with second order phase transitions [32], originally with phase transitions in ferromagnetic materials. It has been applied to various kinds of phase transitions, for instance: the transition nematic/isotropic phase in liquid crystals [33,34,35,36,37,38], or other transitions between liquid crystalline phases [39,40].

Starting with the macroscopic director d, the corresponding alignment tensor is of the form ($\overset{⎴}{AB}$ is the symmetric and traceless part of the tensor AB [41].):

(22) $$a=S\overset{⎴}{dd}=S\left(dd-\frac{1}{3}\delta \right),\;\mathrm{tr}\left(dd\right)=d\cdot d=1,$$

with a scalar quantity, denoted as *Maier–Saupe order parameter* *S*. TheMaier–Saupe order parameter is a measure of the degree of liquid crystalline order, and in equilibrium its value is determined by temperature (and eventually an electric or magnetic field). For the physical interpretation of *S*, we need the mesoscopic background which is treated in Section 4.2.3.

#### 4.1.3. Evolution Equation of the Alignment Tensor

For the exploitation of the dissipation inequality with methods of irreversible thermodynamics [9,31,42], the alignment tensor—but not its gradient—is included in the set of variables. The alignment tensor a(x,t) may vary from continuum element to continuum element, but its gradient does not influence constitutive properties, and therefore it does not appear in the set of variables. This assumption can be looked at as a version of the local equilibrium hypothesis generalized to internal variables. In some situations, no alignment tensor gradient is present at all. For instance, in a nematic liquid crystal between two planar glass plates, with homogeneous boundary conditions and no temperature gradient, the alignment is homogeneous in space [43,44,45].

For the entropy density η and the internal energy density *e*, the following constitutive assumption is made: both quantities are decomposed into a part depending on the equilibrium variables—mass density ϱ and internal energy density *e*—and an alignment tensor dependent part

(23) $$\begin{array}{ccc} \eta & = & \eta_{0}\left(e,\varrho \right)+\eta_{a}\left(a\right) \end{array}$$

(24) $$\begin{array}{ccc} e & = & \epsilon_{0}\left(a=0\right)+\epsilon_{a}\left(a\right). \end{array}$$

For the alignment tensor-independent parts, the Gibbs equation in the usual form holds with pressure *p* and temperature *T*:

(25) $$\frac{d\eta_{0}}{dt}=\frac{1}{T}\frac{d\epsilon_{0}}{dt}-\frac{p}{\varrho^{2}T}\frac{d\varrho }{dt}\;.$$

With the usual assumptions of Thermodynamics of Irreversible Processes concerning the dependence of the entropy flux Φ=q/T on the heat flux q and of the entropy supply φ=r/T on the energy supply *r*, we start out with the balance equation of entropy

(26) $$\sigma =\varrho \frac{d\eta }{dt}+\nabla \cdot \Phi -\varphi .$$

Taking into account the balance equation of the internal energy of a medium with an internal angular momentum Θ·s

(27) $$\varrho \frac{de}{dt}=-\nabla \cdot q+t:\nabla v+r+\varrho \frac{ds}{dt}\cdot \Theta \cdot s$$

(stress tensor: t; material velocity: v; moment of inertia: Θ; and spin density: s), and presupposing a material of vanishing couple stress and couple force

(28) $$\varrho \frac{ds}{dt}=-\epsilon :t,$$

we obtain for the entropy production

(29) $$\begin{array}{c} \sigma =\underset{f_{1}}{\underset{︸}{\varrho \left(\overset{⎴}{\frac{d\eta_{a}}{da}-\frac{1}{T}\frac{d\epsilon_{a}}{da}}\right)}}:\underset{J_{1}}{\underset{︸}{\frac{da}{dt}}}+\underset{J_{2}}{\underset{︸}{q}}\cdot \underset{f_{2}}{\underset{︸}{\left(-\frac{1}{T^{2}}\right)\nabla T}}+\\ +\underset{J_{3}}{\underset{︸}{\frac{1}{T}\left(p+\frac{1}{3}\;\mathrm{trace}\left(t\right)\right)}}\underset{f_{3}}{\underset{︸}{\nabla \cdot v}}+\underset{J_{4}}{\underset{︸}{\frac{1}{T}\overset{⎴}{t}}}:\underset{f_{4}}{\underset{︸}{\overset{⎴}{\nabla v}}}+\\ +\underset{J_{5}}{\underset{︸}{\frac{1}{T}t^{antisym}}}:\underset{f_{5}}{\underset{︸}{\left({\left(\nabla v\right)}^{antisym}-\epsilon :\left(\theta \cdot s\right)\right)}}. \end{array}$$

Linear constitutive relations between the fluxes $J_{1},\cdots ,J_{5}$ and the forces $f_{1},\cdots ,f_{5}$ are considered. It is assumed that the anisotropy of the liquid crystal is given explicitly by the dependence of internal energy and entropy on the alignment tensor, but otherwise material coefficients are scalars. Then, the Curie principle applies, and there is no coupling between fluxes and forces of different tensorial order, and no coupling between symmetric and antisymmetric tensors. With these assumptions, the flux-force-relations read

(30) $$\begin{array}{ccc} \frac{da}{dt} & = & -L_{11}\frac{\varrho }{T}\frac{\overset{⎴}{df_{a}}}{da}+L_{14}\overset{⎴}{\nabla v}, \end{array}$$

(31) $$\begin{array}{ccc} q & = & -\frac{1}{T^{2}}L_{22}\nabla T, \end{array}$$

(32) $$\begin{array}{ccc} \frac{1}{T}\left(p+\frac{1}{3}\;\mathrm{trace}\left(t\right)\right) & = & L_{33}\nabla \cdot v, \end{array}$$

(33) $$\begin{array}{ccc} \overset{⎴}{t} & = & -L_{41}\varrho \frac{\overset{⎴}{df_{a}}}{da}+L_{44}\overset{⎴}{\nabla v}, \end{array}$$

(34) $$\begin{array}{ccc} \frac{1}{T}t^{antisym} & = & L_{55}\left({\left(\nabla v\right)}^{antisym}-\epsilon \cdot \left(\theta \cdot s\right)\right), \end{array}$$

by introducing the anisotropic part of the free energy density

(35) $$f_{a}=\epsilon_{a}-T\eta_{a}.$$

Equation (30) is the evolution equation of the internal variable, the alignment tensor. It is of the form of a pure relaxation equation without a flux term. In the following, the expression in the bracket $\epsilon_{a}-T\eta_{a}=f_{a}$ is abbreviated as the alignment-tensor-dependent part of the free energy density $f_{a}$. The constitutive Equation (31) is the classical Fourier equation with heat conductivity $\kappa =L_{22}/T^{2}$. From (32) follows for vanishing flow field, $p=-\frac{1}{3}\;\mathrm{trace}\left(t\right)$. The remaining two equations are the constitutive relations for the symmetric traceless part of the stress tensor $\overset{⎴}{t}$, and for the antisymmetric part of the stress tensor $t^{antisym}$. To exploit further Equations (30) and (33), expressions for the alignment tensor dependence of $\eta_{a}$ and $\epsilon_{a}$ are needed. We will make constitutive assumptions involving terms up to fourth and second order, respectively:

(36) $$\begin{array}{ccc} \eta_{a}\left(a\right) & = & -\frac{1}{2}A_{0}a:a+\frac{1}{3}B\;\mathrm{trace}\left(a\cdot a\cdot a\right)-\;\\ & & -\frac{1}{4}C_{1}{\left(a:a\right)}^{2}-C_{2}\;\mathrm{trace}\left(a\cdot a\cdot a\cdot a\right), \end{array}$$

(37) $$\begin{array}{ccc} \epsilon_{a}\left(a\right) & = & -\frac{1}{2}\epsilon a:a. \end{array}$$

The coefficients $A_{0},B,C_{1}$, $C_{2}$, and ϵ are material dependent parameters which are assumed to be constant, and, especially independent of temperature. Here, the Cayleigh–Hamilton theorem could be used to transform the expression a·a·a·a, because this is not an independent invariant. However, the above form is the most practical one. The derivations from (36) and (37) yield

(38) $$\begin{array}{ccc} \frac{d\eta_{a}}{da} & = & -A_{0}a+Ba\cdot a-C_{1}a:aa-C_{2}a\cdot a\cdot a, \end{array}$$

(39) $$\begin{array}{ccc} \frac{d\epsilon_{a}}{da} & = & -\epsilon a. \end{array}$$

Using these representations, from (30) the relaxation equation

(40) $$\begin{array}{ccc} \frac{da}{dt} & = & -L_{11}\varrho \overset{⎴}{\frac{1}{T}\frac{df_{a}}{da}}+L_{14}\overset{⎴}{\nabla v}=\\ & = & L_{11}\varrho \left(\overset{⎴}{-A\left(T\right)a+Ba\cdot a-C_{1}a:aa-C_{2}a\cdot a\cdot a}\right)+L_{14}\overset{⎴}{\nabla v} \end{array}$$

follows, with

(41) $$A\left(T\right)=A_{0}-\frac{1}{T}\epsilon .$$

For the symmetric traceless part of the stress tensor, we obtain the constitutive equation:

(42) $$\begin{array}{ccc} \overset{⎴}{t} & = & -L_{41}\varrho \frac{df_{a}}{da}+L_{44}T\overset{⎴}{\nabla v}=\\ & = & L_{41}T\varrho \left(\overset{⎴}{-A\left(T\right)a+Ba\cdot a-C_{1}a:aa-C_{2}a\cdot a\cdot a}\right)+L_{44}T\overset{⎴}{\nabla v}. \end{array}$$

#### 4.1.4. Evolution Equation of the Alignment Tensor without Flow Field

For vanishing velocity field,

(43) v≡0→∇v≡0,

the relaxation equation for the alignment tensor simplifies to

(44) $$\begin{array}{ccc} \frac{da}{dt} & = & -L_{11}\varrho \frac{1}{T}\frac{\overset{⎴}{df_{a}}}{da}=\\ & = & L_{11}\varrho \left(\overset{⎴}{-A\left(T\right)a+Ba\cdot a-C_{1}a:aa-C_{2}a\cdot a\cdot a}\right). \end{array}$$

The right hand side of this equation is proportional to the derivative of a potential, the free energy density $f_{a}$. In other words, for vanishing velocity field, the time derivative of the alignment tensor is governed by a potential. For a non-vanishing velocity gradient, such a derivation from a potential is possible only in very special flow geometries but not in general.

### 4.2. The Mesoscopic Theory

#### 4.2.1. General Remarks

The mesosocpic theory introduces the microscopic director n as a mesoscopic variable, which means the MDF f(m,x,t) (16) becomes the *orientation distribution function* (ODF) f(x,t,n) which describes the orientational distribution of the molecules in the considered volume element of the nematic liquid crystal exactly as points on the 2-dimensional unit sphere $S^{2}$. The drawback is that one must know this distribution function which is not directly measurable. Consequently, approximation methods are necessary for exploiting the advantages of the mesoscopic procedure against the macroscopic one. The ODF has a special property: the head-tail-symmetry, viz.,

(45) f(x,t,n)=f(x,t,−n)≡f(·),

which takes into account that each microscopic director generates two points on the $S^{2}$, one on the “northern hemisphere” and the other is the opposite pole on the “southern hemisphere”. This head-tail-symmetry forbids the interpretation that the macroscopic director describes the mean orientation of the microscopic directors in a particle of the liquid crystal

(46) $$\int_{S^{2}}nf\left(x,t,n\right)d^{2}n=0.$$

Consequently, the question arises: what is the macroscopic director in the framework of the mesoscopic theory?

#### 4.2.2. The Orientation Distribution Function

Thermotropic liquid crystals consist of rigid non-spherical molecules which are rotation symmetric. The axis of this molecular rotation symmetry determines the microscopic director n. The molecules themseves can be rod-like or disc-like. In all liquid crystalline phases, there exists an orientational order of the microscopic directors which is described by the ODF which often has uniaxial symmetry.

**Figure 1:**
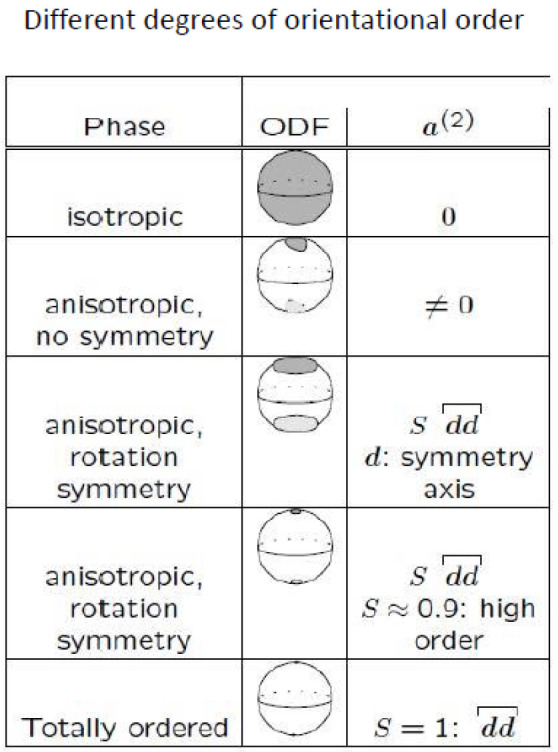
The orientation distribution function (ODF) in the uniaxial and biaxial liquid crystalline phases. In the isotropic phase, all orientations are equally probable, whereas, in the liquid crystalline phases, the ODF is anisotropic.

The ODF allows identification of the different phases. In the isotropic phase, all molecule orientations are equally probable, and the orientation distribution function is isotropic, i.e., a homogeneous function on the unit sphere $S^{2}$. The other extreme is the totally ordered phase, where all molecule orientations are identical. The corresponding distribution function has a non-zero value only for this single common orientation, i.e., it is delta-shaped. Due to the thermal motion, this completely ordered phase does not occur at non-zero temperature. There is a partial ordering of orientations, and the corresponding distribution functions show some concentration around a preferred orientation. There are two possibilities: that the ODF is rotation symmetric around an axis e, or that there is no such rotation symmetry. In the first case, the phase is called uniaxial; in the second case, it is called biaxial (The terms “uniaxial” and “biaxial” are related to the ODF and not to the molecules.). In most cases, nematic liquid crystalline phases are observed to be uniaxial as sketched in Figure 1.

If we denote the angle between the uniaxial symmetry axis by e and a microscopic director n by Θ, the ODF depends only on cosΘ because of this uniaxial symmetry

(47) f(x,t,n)=g(x,t,cosΘ).

The uniaxial symmetry of the ODF causes a special form of the alignment tensor which is discussed in Section 4.2.3.

#### 4.2.3. The Mesoscopic Root of the Alignment Tensor Family

According to (22), the alignment tensor is symmetric, traceless and of second order. Using the ODF and the microscopic director n as a mesoscopic variable, we introduce the family of the macroscopic *fields of order parameters* defined by different moments of the ODF

(48) $$\begin{array}{ccc} a(x,t) & := & \int_{S^{2}}f\left(\cdot \right)\overset{⎴}{nn}\;d^{2}n, \end{array}$$

(49) $$\begin{array}{ccc} a^{\left(4\right)}\left(x,t\right) & := & \int_{S^{2}}f\left(\cdot \right)\overset{⎴}{nnnn}\;d^{2}n, \end{array}$$

(50) $$\begin{array}{ccc} a^{\left(k\right)}\left(x,t\right) & := & \int_{S^{2}}f\left(\cdot \right)\underset{k\;\mathrm{times}}{\underset{︸}{\overset{⎴}{n\cdots n}}}\;d^{2}n,\;etc. \end{array}$$

These tensors are macroscopic fields of successive order. The even order tensors are non-zero, due to the head-tail symmetry of the orientation distribution function (45).

Starting with the uniaxial ODF (47), the alignment tensors of second and higher order become [33]

(51) $$\begin{array}{ccc} a_{unax}\left(x,t\right) & = & S(x,t)\overset{⎴}{e(x,t)e(x,t)},\;e\cdot e=1, \end{array}$$

(52) $$\begin{array}{ccc} a_{unax}^{\left(k\right)}\left(x,t\right) & = & S^{\left(k\right)}\left(x,t\right)\underset{k\;\mathrm{times}}{\overset{⎴}{\underset{︸}{e(x,t)\cdots \cdots e(x,t)}}}. \end{array}$$

Comparison with (22) allows the following interpretation which answers the question posed at the end of Section 4.2.1: the macroscopic director d is defined by the uniaxial symmetry axis e of the ODF.

(53) d(x,t)≡e(x,t).

Apart from this, the following statement is true: If the macroscopic director is a basic field, as in the well-known Ericksen–Leslie theory, all microscopic directors are perfectly aligned along the symmetry axis of the ODF or perpendicular to it [30].

The eigenvalue problems of the alignment tensor of uniaxial ODF are, according to (51) and (53),

(54) $$\begin{array}{ccc} a_{unax}\cdot d & = & S\left(d-\frac{1}{3}d\right)=\frac{2}{3}Sd, \end{array}$$

(55) $$\begin{array}{ccc} a_{unax}\cdot d^{⊥} & = & -S\frac{1}{3}d^{⊥},\;d^{⊥}\cdot d=0. \end{array}$$

The Maier–Saupe parameter becomes a scalar field which can be interpreted mesoscopically:

1. Isotropy (Ordinary liquid phase)
  Each direction is eigenvalue of $a_{unax}$ belonging to the same eigenvalue. According to (54) and (55), we obtain
  (56) $$\frac{2}{3}S=-S\frac{1}{3}\;⟶\;S_{iso}=0\;⟶\;a_{iso}=0.$$
2. Complete alignment (Ericksen–Leslie theory)
  If $d_{tot}$ is the direction of total alignment, the ODF is according to (45)
  (57) $$f_{tot}\left(\cdot \right)=\frac{1}{2}\left(\delta \left(n-d_{tot}\right)+\delta \left(n+d_{tot}\right)\right),$$
  resulting in
  (58) $$\begin{array}{ccc} a_{tot} & = & \int_{S^{2}}\frac{1}{2}\left(\delta (n-d_{tot}+\delta \left(n+d_{tot}\right)\right)\left(nn-\frac{1}{3}\delta \right)d^{2}n=\\ & = & \left(d_{tot}d_{tot}-\frac{1}{3}\delta \right)\;⟶\;S_{tot}=1, \end{array}$$
  according to (51) and (53). Additionally, the scalar order parameters take the value $S_{tot}^{\left(k\right)}=1$.

Consequently, we obtain: the ordinary liquid phase is characterized by S=0 and $a_{iso}=0$, the case S=1 corresponds to the totally ordered phase, where all molecule orientations with respect to the macroscopic director $d_{tot}$ are equal. This is the case for the well-known Ericksen–Leslie theory, where all molecules have exactly the same orientation and all scalar order parameters $S^{\left(k\right)}$ are equal to unity. The value S=−1/2 is the other extreme value (−1/2≤S≤1) which corresponds, according to (55), to a totally ordered planar phase, where all molecule axes n lie in the plane perpendicular to the macroscopic director d. In experiments, partially ordered phases with 0<S<1 are observed.

The fields of order parameters $a^{\left(k\right)}\left(x,t\right)$ describe macroscopically the mesoscopic state of the system introduced by the mesoscopic variable n and its distribution function. Consequently, these fields are the link between the mesoscopic background description of the liquid crystal and its description by additional macroscopic fields as internal variables. In the isotropic phase, all alignment tensors are zero, whereas in the liquid crystalline phases, at least some alignment tensors are non-zero. In equilibrium, they are determined by the equilibrium variables mass density and temperature. The most important one is the alignment tensor of second order (k=2) which is easily measured via optical properties of the liquid crystalline phase.

#### 4.2.4. Evolution Equation of the Alignment Tensor

From the mesoscopic point of view, the equation of motion of the alignment tensor is derived from balance equations of the mesoscopic fields. The orientation distribution function is defined as the mass fraction,

(59) $$f\left(x,t,n\right)=\frac{\rho (x,t,n)}{\rho (x,t)}.$$

The macroscopic mass density ρ(x,t) satisfies the continuity equation, assuming additionally incompressibility. The mesoscopic mass density satisfies, the following balance equation [27,46]

(60) $$\frac{\partial }{\partial t}\varrho \left(\cdot \right)\;+\;\nabla_{x}\cdot \left\{\varrho \left(\cdot \right)v\left(\cdot \right)\right\}+\nabla_{n}\cdot \left\{\varrho \left(\cdot \right)u\left(\cdot \right)\right\}\;=\;0,$$

with the mesoscopic material velocity v(·) and the orientation change velocity u(·) which are defined by

(61) $$\left(x,t,n\right)⟶\;\left(x+v(\cdot )\Delta t,\;t+\Delta t,n+u(\cdot )\Delta t\right).$$

The orientation distribution function satisfies a balance equation because of the definition (59), of the mesoscopic mass balance (60) and of the incompressibility condition. A straight forward calculation results in [41]

(62) $$\frac{\partial f(x,n,t)}{\partial t}+v\left(x,n,t\right)\cdot \nabla f\left(x,n,t\right)+\nabla_{n}\cdot \left(u\left(x,n,t\right)f\left(x,n,t\right)\right)=0.$$

The differential Equation (62) of the ODF allows the derivation of a system of differential equations for the alignment tensors of successive order, after inserting an expression for the orientation change velocity u(·). In these equations, the alignment tensors of all orders may be coupled, depending on the expression for u(·). In general, a closure relation is needed in order to deal with only a limited number of moments (see [47]). A closure relation expresses the higher order alignment tensors $a^{\left(k\right)}\left(x,t\right)$ (k=4,6,⋯) in terms of the second order one. Together with such a closure relation, these equations are the differential equations for the internal variable alignment tensor of second order a(x,t).

### 4.3. Combination of Mesoscopic and Macroscopic Descriptions

A unique reconstruction of the orientation distribution function (59) defined on the higher dimensional mesoscopic space from a macroscopic internal variable is not possible. Only a distribution function in a restricted class of functions can be determined in such a way that the averages calculated with it give the correct value of the internal variables, which are assumed to be known. The class of distribution functions is chosen in such a way, that it maximizes the statistical entropy. This idea of entropy maximization goes back to Jaynes [48,49], and is applied widely in information theory. In the kinetic theory of gases, this principle is applied in order to calculate higher order moments of the velocity distribution [50,51,52,53]. In the context of the mesoscopic theory, it has been applied in [47].

Starting with the ODF maximizing the statistical entropy [47]

(63) $$\begin{array}{ccc} f(x,t,n) & ≐ & \frac{e^{-\Lambda (x,t):\overset{⎴}{nn}}}{\int_{S^{2}}e^{-\Lambda (x,t):\overset{⎴}{nn}}d^{2}n}=:\frac{e^{-\Lambda (x,t):\overset{⎴}{nn}}}{Z}, \end{array}$$

(64) $$\begin{array}{ccc} \Lambda^{⊤} & ≐ & \Lambda ,\;\overset{{}_{{}^{•}}}{\Lambda }:\delta ≐0 \end{array}$$

by use of a symmetric tensor Λ whose time derivative $\overset{{}_{{}^{•}}}{\Lambda }$ is traceless, we obtain for the alignment tensor (48)

(65) $$\begin{array}{ccc} a\left(x,t\right)\;=\;\int_{S^{2}}\overset{⎴}{nn}\frac{e^{-\Lambda (x,t):\overset{⎴}{nn}}}{Z}d^{2}n=\frac{1}{Z}\int_{S^{2}}-\frac{\partial }{\partial \Lambda }e^{-\Lambda :\overset{⎴}{nn}}d^{2}n & = & -\frac{1}{Z}\frac{\partial }{\partial \Lambda }\int_{S^{2}}e^{-\Lambda :\overset{⎴}{nn}}d^{2}n=\\ & = & -\frac{1}{Z}\frac{\partial Z}{\partial \Lambda }=-\frac{\partial \ln Z}{\partial \Lambda }. \end{array}$$

This implicit relation between the alignment tensor and the parameter Λ cannot be solved for Λ. Instead, we will use the entropy density for the identification of Λ.

The part of the entropy density $\eta_{a}$ in (23) which depends only on the alignment tensor is introduced on the microscopic level using the Shannon entropy of the ODF [47]

(66) $$\eta_{a}\left(x,t\right)=K\int_{S^{2}}f\left(x,t,n\right)\ln f\left(x,t,n\right)d^{2}n.$$

Inserting the orientation distribution function (63), this results in

(67) $$\frac{1}{K}\eta_{a}\left(x,t\right)=\int_{S^{2}}-\Lambda \left(x,t\right):\overset{⎴}{nn}f\left(x,t,n\right)\;d^{2}n-\ln Z=-\Lambda :a-\ln Z.$$

Taking

(68) $$\overset{{}_{{}^{•}}}{Z}=\frac{d}{dt}\left(\int_{S^{2}}e^{-\Lambda (x,t):\overset{⎴}{nn}}d^{2}n\right)=-\overset{{}_{{}^{•}}}{\Lambda }:\int_{S^{2}}e^{-\Lambda (x,t):\overset{⎴}{nn}}\;\overset{⎴}{nn}d^{2}n=-\overset{{}_{{}^{•}}}{\Lambda }:aZ$$

into account, we obtain according to (65)

(69) $${\left(\ln Z\right)}^{{}^{•}}=-\overset{{}_{{}^{•}}}{\Lambda }:a=\overset{{}_{{}^{•}}}{\Lambda }:\frac{\partial \ln Z}{\partial \Lambda }.$$

The LHS of (69) is a total differential. Consequently, according to (69)${}_{2}$, lnZ depends only on Λ. Because lnZ is a scalar under observer changes (frame independence), its dependence on Λ is via its scalar invariants [54,55]. Here, we choose a simple case

(70) $$\begin{array}{ccc} \ln Z & ≐ & g\left(\Lambda -\frac{1}{3}\left(\mathrm{tr}\Lambda \right)\delta \right):\left(\Lambda -\frac{1}{3}\left(\mathrm{tr}\Lambda \right)\delta \right)=g\left(\Lambda :\Lambda -\frac{1}{3}{\left(\mathrm{tr}\Lambda \right)}^{2}\right), \end{array}$$

(71) $$\begin{array}{cc} & \frac{\partial g}{\partial t}≐0,\;\frac{\partial g}{\partial \Lambda }≐0. \end{array}$$

Taking (64) into account, we obtain from (70) and (69)

(72) $${\left(\ln Z\right)}^{{}^{•}}=g\left(2\overset{{}_{{}^{•}}}{\Lambda }:\Lambda -\frac{2}{3}\left(\mathrm{tr}\Lambda \right){\left(\mathrm{tr}\Lambda \right)}^{{}^{•}}\right)=2g\overset{{}_{{}^{•}}}{\Lambda }:\Lambda =-\overset{{}_{{}^{•}}}{\Lambda }:a.$$

Because of (64), we can identify from (72)

(73) $$2g\left(\Lambda -\frac{1}{3}\left(\mathrm{tr}\Lambda \right)\delta \right)≐-a\;⟶\;\Lambda =-\frac{1}{2g}a+\frac{1}{3}\left(\mathrm{tr}\Lambda \right)\delta .$$

Taking (73) and (70) into consideration, we obtain

(74) $$\begin{array}{ccc} -\Lambda :\overset{⎴}{nn} & = & \left(\frac{1}{2g}a-\frac{1}{3}\left(\mathrm{tr}\Lambda \right)\delta \right):\overset{⎴}{nn}=\frac{1}{2g}a:\overset{⎴}{nn}-\frac{1}{3}\left(\mathrm{tr}\Lambda \right)\left(\mathrm{tr}\overset{⎴}{nn}\right)=\frac{1}{2g}a:\overset{⎴}{nn},\; \end{array}$$

(75) $$\begin{array}{ccc} \ln Z & = & g\left(-\frac{1}{2g}a+\frac{1}{3}\left(\mathrm{tr}\Lambda \right)\delta \right):\left(-\frac{1}{2g}a+\frac{1}{3}\left(\mathrm{tr}\Lambda \right)\delta \right)-\frac{g}{3}{\left(\mathrm{tr}\Lambda \right)}^{2}=\frac{1}{4g}a:a. \end{array}$$

Taking (74) into account, the ODF (63) becomes

(76) $$f\left(x,t,n\right)=\frac{1}{Z}\exp\left(\frac{1}{2g}a\left(x,t\right):\overset{⎴}{nn}\right),\;Z=\int_{S^{2}}\exp\left(\frac{1}{2g}a\left(x,t\right):\overset{⎴}{nn}\right)d^{2}n,$$

and the alignment tensor (65) yields

(77) $$a\left(x,t\right)=\frac{1}{Z}\int_{S^{2}}\overset{⎴}{nn}\exp\left(\frac{1}{2g}a\left(x,t\right):\overset{⎴}{nn}\right)d^{2}n.$$

The entropy density (67) becomes by use of (73) and (75)

(78) $$\frac{1}{K}\eta_{a}\left(a\right)=\left(\frac{1}{2g}a-\frac{1}{3}\left(\mathrm{tr}\Lambda \right)\delta \right):a-\frac{1}{4g}a:a=\frac{1}{4g}a:a.$$

By the choice (70)—which was induced by frame independence—we obtained (78), the quadratic dependence of the entropy density on the alignment tensor. This simple expression is often a too rough approximation: the quadratic term has to be extended by terms of higher order which must also be scalar invariants according to observer independence.

## 5. Further Applications of Mesoscopic Theory

The mesoscopic concept has been applied to various kinds of materials with an internal structure, like solids damaged by micro-cracks [56,57,58,59,60,61,62,63], dipolar media [64], mixtures [65,66], granular materials [67], magnetorheological fluids [68] and fiber reinforced concrete [69,70]. Three different applications will be sketched in the following.

### 5.1. Solids, Damaged by Micro-Cracks

An important mechanism of material damage in solids is the growth of micro-cracks under the action of an external load. These microcracks can be modelled as penny-shaped, i.e., flat and rotation symmetric. Then, each single crack is characterized by its diameter and orientation of the surface normal [57,58,60]. In the case of microscopically small cracks, there is a large number of cracks in the volume element with a distribution of crack sizes and crack orientations. The crack length may take values between a minimum length $l_{m}$ of the smallest preexisting cracks and a maximum length $l_{M}$, which is limited by the linear dimension of the continuum element. The orientation of the unit vector n is given by an element of the unit sphere $S^{2}$. Therefore, in the example of microcracks the manifold M of the mesoscopic variables is given by $\left[l_{m},l_{M}\right]\times S^{2}$.

#### 5.1.1. Definition of the Crack Distribution Function

Due to its definition as probability density, the crack distribution function (CDF) is the number fraction

(79) $$f\left(l,n,x,t\right)=\frac{N(l,n,x,t)}{N(x,t)},$$

in volume elements for which the number density N(x,t) is non-zero. Here, N(x,t) is the macroscopic number density of cracks of any length and orientation. If N(x,t)=0, we define additionally that in this case f(l,n,x,t)≐0. As there is no creation of cracks in our model, the distribution function will be zero for all times in these volume elements. In all other volume elements with a non-zero crack number, the normalization

(80) $$\int_{l_{m}}^{l_{M}}\int_{S^{2}}f\left(l,n,x,t\right)l^{2}d^{2}ndl=1$$

is used.

#### 5.1.2. Balance of Crack Number

In our model, the cracks move together with the material element. Consequently, their flux is the convective flux, having a part in position space, a part in orientation space and a part in the length interval. There is no production and no supply of the crack number. Therefore, we have for the crack number density *N*

(81) $$\frac{\partial }{\partial t}N\left(\cdot \right)+\nabla_{x}\cdot \left\{N\left(\cdot \right)v\left(x,t\right)\right\}+\nabla_{n}\cdot \left\{N\left(\cdot \right)u\left(x,t\right)\right\}+\frac{1}{l^{2}}\frac{\partial }{\partial l}\left(l^{2}\dot{l}N\left(\cdot \right)\right)\;=\;0.$$

We obtain a balance of the CDF (79) by inserting N(·) into (81). This yields

(82) $$\begin{array}{c} \frac{\partial }{\partial t}f\left(l,n,x,t\right)+\nabla_{x}\cdot \left(v(x,t)f(l,n,x,t)\right)+\;\\ +\nabla_{n}\cdot \left(u(x,t)f(l,n,x,t)\right)+\frac{1}{l^{2}}\frac{\partial }{\partial l}\left(l^{2}\dot{l}f\left(l,n,x,t\right)\right)=\\ =\frac{-f(l,n,x,t)}{N(x,t)}\left(\frac{\partial }{\partial t}+v\left(x,t\right)\cdot \nabla_{x}\right)N\left(x,t\right)=\\ =\frac{-f(l,n,x,t)}{N(x,t)}\frac{dN(x,t)}{dt}=0. \end{array}$$

This balance equation of the CDF corresponds to that of the ODF (62) in liquid crystal theory.

#### 5.1.3. Definition of a Damage Parameter

The damage parameter is introduced as a macroscopic quantity growing with progressive damage in such a way that it should be possible to relate the change of material properties to the growth of the damage parameter. We define the damage parameter as the fraction of cracks, which have reached a certain length *L*. The idea is that cracks of this and larger sizes considerably decrease the strength of the material, and, therefore, their fraction is a measure of damage. This idea is related to the slender bar model of Krajcinovic [71], where the damage parameter is introduced as the number of “broken bars” in the sample,

(83) $$D\left(x,t\right)=\int_{L}^{\infty }\int_{S^{2}}f\left(l,n,x,t\right)d^{2}nl^{2}dl.$$

In this definition of the damage parameter the possibility of cracks of any length ($l_{M}\to \infty$) is included. This is consistent with different laws of crack growth, where the crack does not stop growing.

#### 5.1.4. Differential Equation for the Damage Parameter

Differentiating the definition of the damage parameter (83) with respect to time, the following differential equation is obtained,

(84) $$\begin{array}{c} \frac{dD(x,t)}{dt}=-{\left[l^{2}f\left(l,n,x,t\right)\dot{l}\right]}_{L}^{l_{M}}+2\int_{L}^{l_{M}}\int_{S^{2}}f\left(l,n,x,t\right)l\dot{l}d^{2}ndl. \end{array}$$

The differential equation of the damage parameter depends on the crack distribution function, and, consequently, on the initial crack distribution. Additionally, the time rate of change of the damage parameter (84) depends on the differential equation of the crack length.

#### 5.1.5. Closing the Differential Equation of the Crack Distribution Function

Some model on the growth velocity of a single crack is needed in order to generate a closed differential equation for the length and orientation distribution function according to (82). We suppose that, for a given load, not all cracks start growing, but only cracks exceeding a certain critical length $l_{c}$, which is given by the Griffith-criterion. As in many examples of dynamics of crack-length change, the cracks do not stop growing, but extend indefinitely. In all these cases, the maximum crack length has to be set equal to $l_{M}=\infty$. However, when the cracks become macroscopic, their growth dynamics becomes more complicated than our example dynamics here (showing for instance branching).

#### 5.1.6. Onset of Growth: Griffith-Criterion

The criterion for cracks to start growing adopted in the example is the energy criterion introduced originally by Griffith [72]. According to that, there is a criticality condition for the crack growth to start, and for cracks larger than a critical length there is a velocity of crack growth $\dot{l}$. From energetic considerations, Griffith [72] derived a critical length of cracks so that cracks exceeding this length start to grow. This critical length is given by

(85) $$l_{c}=\frac{K}{\sigma_{n}^{2}},$$

where *K* is a material constant, and $\sigma_{n}$ is the stress applied perpendicular to the crack surface. It is assumed that a stress component within the crack plane does not cause crack growth. For cracks smaller than the critical length $l_{c}$, the energy necessary to create the crack surface exceeds the energy gain due to release of stresses.

#### 5.1.7. Rice–Griffith Dynamics

An example of crack dynamics, taking into account the criticality condition of Griffith is derived from a generalization of the Griffith energy criterion on thermodynamic grounds, by introducing a Gibbs potential which includes the stress normal to the crack surface and the crack length as variables. The resulting crack evolution law has the form

(86) $$\begin{array}{ccc} \dot{l}=-\alpha +\beta \sigma^{2}l & \mathrm{for} & \;l\geq l_{c}\;, \end{array}$$

(87) $$\begin{array}{ccc} \dot{l}=0 & \mathrm{for} & \;l<l_{c}, \end{array}$$

with material coefficients α and β. In the case of a constant time rate of the applied stress, $\sigma =v_{\sigma }t$, it results in

(88) $$\begin{array}{ccc} \dot{l}=-\alpha +\beta v_{\sigma }^{2}lt^{2} & \mathrm{for} & \;l\geq l_{c}, \end{array}$$

(89) $$\begin{array}{ccc} \dot{l}=0 & \mathrm{for} & \;l<l_{c}. \end{array}$$

$v_{\sigma }$ is the time derivative of the applied stress normal to the crack surface. The dependence of this normal stress on the crack orientation results in the following orientation dependence of the dynamics

(90) $$\begin{array}{ccc} \dot{l}=-\alpha +\beta v_{\sigma 0}^{2}lt^{2}{\left(e_{z}\cdot n\right)}^{4} & \mathrm{for} & \;l\geq l_{c}, \end{array}$$

(91) $$\begin{array}{ccc} \dot{l}=0 & \mathrm{for} & l<l_{c}, \end{array}$$

where $v_{\sigma 0}$ is the change velocity of the stress applied in the *z*-direction. After averaging over all orientations this orientation dependence results in a dependence on the fourth moment $\int_{S^{2}}nnnnfd^{2}n$ of the crack distribution function. This dynamics also includes a criticality condition for starting the crack growing.

With this model for the velocity of the length change, we end up with the following differential equation for the crack distribution function

(92) $$\begin{array}{ccc} \frac{df(l,n,x,t)}{dt}=-\frac{1}{l^{2}}\frac{\partial }{\partial l}\left(l^{2}\left(-\alpha +\beta v_{\sigma }{\left(n\right)}^{2}lt^{2}\right)\right) & \mathrm{for} & l\geq l_{c}, \end{array}$$

(93) $$\begin{array}{ccc} \frac{df(l,n,x,t)}{dt}=0 & \mathrm{for} & l<l_{c}. \end{array}$$

**Figure 2:**
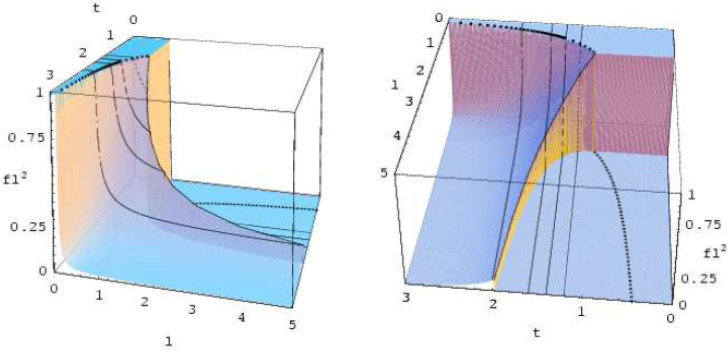
Time evolution for the crack-length distribution function for stepwise initial condition.

Solutions of this differential equation for different initial conditions have been discussed in [60]. An example taken from [60] is depicted in Figure 2.

### 5.2. Dipolar Media

Let us denote the orientation of a single dipole by a unit vector n. The orientation of the dipole can take any value on the unit sphere $S^{2}$. According to the concept of the mesoscopic theory, we introduce mesoscopic fields, defined on the mesoscopic space $R_{x}^{3}\times R_{t}\times S^{2}$. The last argument in the domain of the fields is the orientation of the dipole n. This mesoscopic space is the same as for liquid crystals, and, consequently, the mesoscopic balance equations look the same for a dipolar medium as for liquid crystals. The difference between these two materials is evident in the constitutive theory. An important difference is that the head-tail-symmetry (45) of liquid crystals does *not* exist for dipoles because the dipole and its reverse are distinguishable.

#### 5.2.1. Orientation Distribution Function and Alignment Tensors

Macroscopically, the dipole moments manifest themselves as a magnetization only if their orientations are not distributed isotropically, but they are oriented more or less in parallel. This orientational order can be described by using the orientation distribution function (ODF) of the liquid crystal theory which is sketched in Section 4.2.4. Thus the definition of the ODF (59) and its balance Equation (62) are also valid for dipolar media, except for the head-tail-symmetry (45). In addition, the alignment tensor family (48)–(50) mentioned in Section 4.2.3 is identical for (nematic) liquid crystals and dipolar media. In contrast to the vanishing first order alignment tensor (46) of the liquid crystal theory, it is here proportional to the macroscopic magnetization.

It is convenient to also introduce alignment tensors $A^{\left(k\right)}$ which are not traceless,

(94) $$A^{\left(k\right)}\left(x,t\right):=\int_{S^{2}}f\left(x,n,t\right)\underset{k}{\underset{︸}{n\cdots n}}\;d^{2}n.$$

#### 5.2.2. Exploitation of the Spin Balance Equation

The domain of the mesoscopic constitutive mappings—the state space *Z*—ischosen to be

(95) $$\begin{array}{c} Z=\{\varrho ,T,B,\dot{B},a^{\left(1\right)},a^{\left(2\right)},n\}. \end{array}$$

Here, *T* is the temperature and B the magnetic induction. The state space includes macroscopic and mesoscopic variables. The macroscopic variables are temperature, mass density, magnetic induction, its time derivative, and the first and second order alignment tensors. These alignment tensors in the state space account for the fact that the dipoles tend to align in parallel, i.e., the surrounding dipoles exert an aligning “mean field”.

In a simpler model, it would be sufficient to include only the first order alignment tensor which expresses the tendency of the dipoles to align in parallel. The second order alignment tensor accounts for the influence of a quadrupolar ordering. We will discuss the case without the second order alignment tensor as a special case later. The mass density ϱ in the state space is the macroscopic one because the dependence on the orientation n is written out explicitly.

An exploitation of the balance of spin together with a constitutive function for the stress tensor results in the orientation change velocity

(96) $$\begin{array}{c} u=\left(\delta -nn\right)\cdot \left(\beta_{1}B+\beta_{2}\dot{B}+\beta_{4}a^{\left(1\right)}+\beta_{3}a^{\left(2\right)}\cdot n\right). \end{array}$$

The coefficients $\beta_{j}$ are functions of the macroscopic mass density ϱ(x,t) and the temperature T(x,t).

#### 5.2.3. Equation of Motion of the Magnetization

The first moment of Equation (62) reads

(97) $$\frac{\partial }{\partial t}\int_{S^{2}}fnd^{2}n+v\cdot \nabla \int_{S^{2}}fnd^{2}n+\int_{S^{2}}n\cdot \nabla_{n}\left(fu\right)d^{2}n=0.$$

On the other hand, the variable n is proportional to the microscopic magnetization (magnetization per unit mass), i.e., it is the orientation of the microscopic dipole moment: m=αn with α=const. The first moment of the orientation distribution function is proportional to the average of the microscopic magnetization, i.e., the macroscopic magnetization

(98) $$M\left(x,t\right)=\alpha \varrho \left(x,t\right)\int_{S^{2}}fnd^{2}n=\alpha \varrho \left(x,t\right)a^{\left(1\right)}.$$

The first two terms in Equation (97) are derivatives of the first order alignment tensor. The third term is integrated by parts using Gauss’ theorem on the unit sphere. The resulting equation reads

(99) $$\frac{\partial a^{\left(1\right)}}{\partial t}+v\cdot \nabla a^{\left(1\right)}=\frac{da^{\left(1\right)}}{dt}=\int_{S^{2}}fu\cdot \nabla_{n}\left(n\right)d^{2}n.$$

Then, inserting the equation for the orientation change velocity, Equation (96), and taking into account $\nabla_{n}\left(n\right)=P=\delta -nn$ and $n\cdot \nabla_{n}\left(\cdots \right)=0$ (because $\nabla_{n}$ is the covariant derivative on the unit sphere), one obtains

(100) $$\begin{array}{c} \frac{da^{\left(1\right)}}{dt}=\int_{S^{2}}\left(\beta_{1}B+\beta_{2}\dot{B}+\beta_{4}a^{\left(1\right)}+\beta_{5}a^{\left(2\right)}\cdot n\right.-\\ \left.-\beta_{1}nn\cdot B-\beta_{2}nn\cdot \dot{B}-\beta_{4}nn\cdot a^{\left(1\right)}\right)fd^{2}n, \end{array}$$

in which the fact that P is a projector (P·P=P) has been used.

The first moment of the dipole distribution function is proportional to the magnetization (see Equation (98)). In the resulting equation, there enters also the second orientational moment $A^{\left(2\right)}$ of the dipole distribution function, viz.,

(101) $$\begin{array}{ccc} A^{\left(2\right)} & = & \int_{S^{2}}f\left(\cdot \right)nnd^{2}n\;. \end{array}$$

For an incompressible material, we find

(102) $$\begin{array}{c} \frac{1}{\alpha \varrho }\frac{dM}{dt}=\beta_{1}B+\beta_{2}\dot{B}+\beta_{4}\frac{1}{\alpha \varrho }M-\;\\ -\beta_{1}A^{\left(2\right)}\cdot B-\beta_{2}A^{\left(2\right)}\cdot \dot{B}-\beta_{4}\frac{1}{\alpha \varrho }A^{\left(2\right)}\cdot M. \end{array}$$

A closure relation is needed, expressing the higher order moments in terms of the second order one. Such a closure relation can be derived from the principle of maximum entropy [47], or it has to be postulated as a constitutive equation. The simplest assumption is that the orientations of the dipoles are statistically independent (which is an approximation only). Then, the closure relation is a very simple as follows:

(103) $$\begin{array}{c} A^{\left(2\right)}=\int_{S^{2}}f\left(x,n,t\right)nnd^{2}n=\int_{S^{2}}f\left(x,n,t\right)nd^{2}n\int_{S^{2}}f\left(x,n,t\right)nd^{2}n=\\ =a^{\left(1\right)}a^{\left(1\right)}, \end{array}$$

(104) $$\begin{array}{c} \frac{dM}{dt}=\beta_{1}\alpha \varrho B+\beta_{2}\alpha \varrho \dot{B}+\beta_{4}M-\;\\ -\beta_{1}\frac{1}{\alpha \varrho }MM\cdot B-\beta_{2}\frac{1}{\alpha \varrho }MM\cdot \dot{B}-\beta_{4}\frac{1}{\alpha^{2}\varrho^{2}}MM\cdot M. \end{array}$$

If the value of the magnetization is sufficiently small, we can neglect quadratic and higher order terms of the magnetization. In this linear limit, (104) simplifies to

(105) $$\begin{array}{c} \frac{dM}{dt}=\beta_{1}\alpha \varrho B+\beta_{2}\alpha \varrho \dot{B}+\beta_{4}M. \end{array}$$

This expression is of the form of the well-known Debye equation for dielectric relaxation phenomena, here in an analogous form for magnetic relaxation. This fact can be used to identify the coefficients $\beta_{1}$, $\beta_{2}$, and $\beta_{4}$.

### 5.3. Suspensions of Flexible Fibers

#### 5.3.1. Deformation of a Fiber

The fibers will be assumed to be straight, if not loaded. Then, one can choose a coordinate *s* along this fiber orientation and an orthogonal tensor U(s) describing the distortion of the fiber. The angular distortion tensor, defined by

(106) $$\varphi :=U^{T}\cdot \frac{dU}{ds},$$

takes into account the local deformation of the flexible fibers. *s* is the local coordinate along the fiber, and x is the position of the continuum element. The variable *s* is only introduced to describe the local fiber deformation. The tensor φ is obviously skew-symmetric because U is orthogonal.

We introduce the angular distortion vector (the vector invariant of the angular distortion tensor) as

(107) $$\vec{\varphi }\times \delta =\varphi .$$

In this case, however, we will denote the vector by the symbol $\vec{\;}\;$ to distinguish it from the tensor φ.

Let **n** denote the unit vector tangential to the undeformed fiber. The scalar product of $\vec{\varphi }$ and **n** results in the twist

(108) $$t=\vec{\varphi }\cdot n,$$

and the component of $\vec{\varphi }$ perpendicular to **n** is the bend

(109) $$b=\vec{\varphi }-n\left(n\cdot \vec{\varphi }\right).$$

The element of internal structure in our example is the orientation and deformation of the fiber. The orientation of an undeformed fiber is described by a unit vector **n**, where turning around the fiber by π does not change the orientation and therefore n→−n is a symmetry transformation. The vector **n** is an element of the unit sphere $S^{2}$. The deformation of the fiber is given by the vector $\vec{\varphi }$ introduced previously.

#### 5.3.2. Orientational Order Parameter and Deformation Variable

The aim is to introduce macroscopic quantities from this mesoscopic background which describe the distribution of fiber orientations and the average distortion of fibers.

Orientational order parameters:

(110) $$A^{k}=\int_{S^{2}}\int_{R^{3}}f\left(\vec{\varphi },n,x,t\right)\underset{k}{\underset{︸}{n\cdots n}}d^{3}\varphi d^{2}n,$$

Deformation order parameters:

(111) $$\Phi^{k}=\int_{S^{2}}\int_{R^{3}}f\left(\vec{\varphi },n,x,t\right)\underset{k}{\underset{︸}{\vec{\varphi }\cdots \vec{\varphi }}}d^{3}\varphi d^{2}n.$$

Mixed orientation-deformation parameters:

(112) $$\begin{array}{c} a^{m}{\vec{\phi }}^{n}=\int_{S^{2}}\int_{R^{3}}f\left(\vec{\varphi },n,x,t\right)\underset{m}{\underset{︸}{n\cdots n}}\underset{n}{\underset{︸}{\vec{\varphi }\cdots \vec{\varphi }}}d^{3}\varphi d^{2}n. \end{array}$$

These order parameters are tensors of successive orders. They are macroscopic fields depending on position and time. With respect to fiber orientations, we have the symmetry transformation n→−n. Therefore, all odd order orientational order parameters vanish, and the first non-zero order parameter, apart from the isotropic part $A^{0}=1$ is of second order: $A=A^{2}$.

#### 5.3.3. Mesoscopic and Macroscopic Stress Tensor

In the case that all fibers have the same translational velocity, the macroscopic stress tensor is the integral over all mesoscopic ones,

(113) $$t=\int_{S^{2}}\int_{R^{3}}\hat{t}\left(\cdot \right)d^{3}\varphi d^{2}\hat{n}.$$

The mesoscopic stress tensor is a constitutive quantity, defined on a suitable set of variables. This set of variables may include mesoscopic as well as macroscopic quantities. A reasonable choice for this set of variables is

(114) $$\hat{Z}=\left\{\rho ,T,n,\vec{\varphi },\overset{⎴}{\nabla v},\nabla \times v\right\}.$$

Using this set of variables and a representation theorem up to linear order in the velocity gradient and the deformation variable $\vec{\varphi }$, one obtains the following expression for the mesoscopic stress tensor

(115) $$\begin{array}{c} \hat{t}=\frac{\hat{\rho }}{\rho }\left(\alpha_{1}nn+\alpha_{2}n\vec{\varphi }+\alpha_{3}\vec{\varphi }n+\alpha_{4}n\left(\nabla \times v\right)+\alpha_{5}\left(\nabla \times v\right)n+\alpha_{6}\overset{⎴}{\nabla v}+\right.\\ \left.+\alpha_{7}nn\cdot \overset{⎴}{\nabla v}+\alpha_{8}n\cdot \overset{⎴}{\nabla v}n+\alpha_{9}n\cdot \overset{⎴}{\nabla v}\cdot nnn\right). \end{array}$$

The material coefficients $\alpha_{1}$ to $\alpha_{9}$ may all depend on the (macroscopic) mass density ρ and temperature *T*.

We assume, that the material velocity v does not depend on fiber orientation or fiber deformation. In this case, the stress tensor is obtained by averaging over the mesoscopic variables according to Equation (113). This process yields

(116) $$\begin{array}{c} t=\int_{S^{2}}\int_{R^{3}}\frac{\hat{\rho }}{\rho }\left(\alpha_{1}nn+\alpha_{2}n\vec{\varphi }+\alpha_{3}\vec{\varphi }n+\alpha_{4}n\left(\nabla \times v\right)+\alpha_{5}\left(\nabla \times v\right)n+\;\right.\\ \left.+\alpha_{6}\overset{⎴}{\nabla v}+\alpha_{7}nn\cdot \overset{⎴}{\nabla v}+\alpha_{8}n\cdot \overset{⎴}{\nabla v}n+\alpha_{9}n\cdot \overset{⎴}{\nabla v}\cdot nnn\right)d^{3}\varphi d^{2}n=\\ =\alpha_{1}A+\alpha_{2}\langle n\varphi \rangle +\alpha_{3}\langle \varphi n\rangle +\alpha_{6}\overset{⎴}{\nabla v}+\;\\ +\alpha_{7}A\cdot \overset{⎴}{\nabla v}+\alpha_{8}\overset{⎴}{\nabla v}\cdot A+\alpha_{9}\overset{⎴}{\nabla v}:A^{\left(4\right)}. \end{array}$$

The average of $\alpha_{4}n\left(\nabla \times v\right)$ vanishes, because $\int_{S^{2}}fnd^{2}n=0$ due to the symmetry n↔−n, analogously for the term with $\alpha_{5}$. The averages $\langle n\vec{\varphi }\rangle$ and $\langle \vec{\varphi }n\rangle$ are non-zero, because they are even functions of n

(117) φ(−n)=−φ(n),

and therefore

(118) $$-n\vec{\varphi }\left(-n\right)=n\vec{\varphi }\left(n\right).$$

The stress tensor (116) clearly may have an antisymmetric part $t^{a}$

(119) $$t^{a}=\frac{1}{2}\left(\alpha_{2}-\alpha_{3}\right)\left(\langle n\vec{\varphi }\rangle -\langle \vec{\varphi }n\rangle \right)+\frac{1}{2}\left(\alpha_{7}-\alpha_{8}\right)\left(A\cdot \overset{⎴}{\nabla v}-\overset{˚}{d}\cdot A\right)$$

which indicates that the spin balance equation of the material in consideration does not vanish identically.

## 6. Discussion

Constitutive equations of complex materials require a domain which is extended in comparison to that of hydrodynamics. These additional variables are macroscopic fields defined on space–time, often internal variables—“measurable, but not contollable”. There are two cases: these additional variables are basic fields, which means they are entities of their own, or there exists a microscopic background which allows to derive these additional variables. These two possibilities are discussed using the (macroscopic) director and the alignment tensor of nematic liquid crystals.

The microscopic background can be quantum-theoretical, statistical or mesoscopic which is chosen here. Mesoscopic means the domain of space–time is extended by so-called mesoscopic variables to each of them a mesoscopic distribution function (MDF) belongs describing the distribution of the mesoscopic variable in a volume element around the space–time event.

The mesoscopic tools of the nematic liquid crystal theory are the *microscopic director* describing the alignment of each molecule in the considered volume element and the corresponding *orientation distribution function* (ODF) describing their alignment distribution.

The Ericksen–Leslie theory [7,8] introduces the *macroscopic director* as a basic field. This means mesoscopically investigated, locally total or planar alignment of all molecules [30]. If the ODF is uniaxial, the *alignment tensor* has the Maier–Saupe form (22). The Hess theory [42] introduces the alignment tensor as a basic field inducing that the ODF may be arbitrary.

The advantage of the mesoscopic description is not only to interpret the macroscopic quantities, but also to reflect the phase transition liquid-nematic. The shape of the mesoscopic balance equations is well known from mixture theory, including the evolution equation of the MDF.
